# Pro-Apoptotic and Cytotoxic Effects of Melittin on HL-60 Acute Promyelocytic Leukemia Cells: Implications for Retinoid-Independent Therapy

**DOI:** 10.3390/molecules30204093

**Published:** 2025-10-15

**Authors:** Maksymilian Stela, Michał Ceremuga, Natalia Cichon, Tomasz Poplawski, Marcin Podogrocki, Leslaw Gorniak, Michał Bijak

**Affiliations:** 1 Biohazard Prevention Centre, Faculty of Biology and Environmental Protection, University of Lodz, Pomorska 141/143, 90-236 Lodz, Poland; maksymilian.stela@biol.uni.lodz.pl (M.S.); marcin.podogrocki@biol.uni.lodz.pl (M.P.); leslaw.gorniak@biol.uni.lodz.pl (L.G.); michal.bijak@biol.uni.lodz.pl (M.B.); 2 Military Institute of Armoured and Automotive Technology, Okuniewska 1, 05-070 Sulejówek, Poland; michal.ceremuga@witpis.eu; 3 Department of Microbiology and Pharmaceutical Biochemistry, Medical University of Lodz, Mazowiecka 5, 92-215 Lodz, Poland; tomasz.poplawski@umed.lodz.pl

**Keywords:** acute promyelocytic leukemia, HL-60 cell line, melittin, bee venom, apoptosis, mitochondrial pathway, caspase activation, natural compounds

## Abstract

Background: Acute promyelocytic leukemia (APL) is a subtype of acute myeloid leukemia driven by the PML/RARα fusion protein. Standard treatment with all-trans retinoic acid (ATRA) combined with chemotherapy is effective, but resistance and adverse effects remain significant challenges. Melittin, the primary peptide component of bee venom, has demonstrated potent anticancer activity across multiple leukemia subtypes through mitochondrial-dependent mechanisms. Building upon this established evidence, we investigated melittin’s therapeutic potential in APL to address the specific clinical challenge of ATRA resistance. Methods: The cytotoxic and pro-apoptotic effects of melittin were studied on the human APL cell line HL-60. Cell viability was assessed using MTT and trypan blue assays. Mitochondrial membrane potential (MMP) was measured with JC-1 staining. Apoptosis was quantified using Annexin V/propidium iodide flow cytometry, caspase-3/7 activity assays, and real-time PCR analysis of apoptosis-related genes (BCL-2, BAX, APAF-1, CASP-3, CASP-8, CASP-9). Results: Melittin reduced HL-60 cell viability in a dose- and time-dependent manner, with significant decreases after 24 and 48 h. MMP analysis revealed mitochondrial depolarization, and Annexin V staining confirmed the induction of apoptosis. Caspase-3/7 activity increased markedly, supporting activation of the intrinsic apoptotic pathway. Gene expression profiling revealed downregulation of the anti-apoptotic BCL-2 and upregulation of the pro-apoptotic BAX, APAF1, and CASP3. At the same time, CASP8 and CASP9 showed no significant changes, suggesting a predominant involvement of the intrinsic pathway. Conclusions: These findings confirm and extend established evidence by demonstrating that melittin’s mitochondrial apoptotic mechanism is consistently active in promyelocytic HL-60 model (PML/RARα-negative). This retinoid-independent mechanism suggests potential therapeutic utility for ATRA-resistant cases or as a complementary strategy in APL treatment. However, selectivity validation in non-cancerous hematopoietic cells represents an important future research priority.

## 1. Introduction

Acute promyelocytic leukemia (APL) is a specific type of acute myeloid leukemia (AML) characterized by a chromosomal translocation, t(15;17)(q24;q21), which results in the PML/RARα fusion protein [1,2,3]. This abnormal transcription factor inhibits promyelocyte differentiation and encourages their uncontrolled proliferation, leading to the buildup of immature blood cells in the bloodstream and bone marrow [4,5]. APL makes up approximately 5–30% of new adult AML cases and is associated with distinctive clinical features, such as severe bleeding caused by disseminated intravascular coagulation (DIC) and low platelet counts [6,7].

The introduction of all-trans retinoic acid (ATRA) revolutionized APL treatment by directly targeting the PML/RARα fusion protein. ATRA binds to this abnormal receptor, prompting malignant promyelocytes to differentiate into mature neutrophils [8,9]. The current standard approach combines ATRA with anthracycline-based chemotherapy, achieving complete remission rates of 70–75% [10,11]. However, despite these notable advances, significant therapeutic challenges remain, emphasizing the need to explore alternative treatments.

Three major limitations of current APL therapy highlight the need for more treatment options. First, retinoic acid syndrome (RAS) affects 10–30% of patients and is marked by fever, respiratory distress, weight gain, and potentially serious complications [12,13]. Second, resistance to ATRA arises through mechanisms such as increased cytochrome P450-mediated metabolism, higher levels of cellular retinoid-binding proteins, and mutations in the RARα ligand-binding domain of the PML/RARα fusion protein [14,15]. Third, the toxicity associated with intensive chemotherapy limits treatment choices, especially for elderly patients or those with other health issues [16].

Both RAS and ATRA resistance are closely connected to the retinoid receptor pathway, leading to a significant treatment gap. Patients who become resistant to or develop complications with ATRA-based therapy lack effective options that completely bypass this signaling system. As a result, drugs that operate through mechanisms independent of retinoid signaling are emerging as promising alternatives for APL. These could benefit ATRA-resistant patients or be combined with other treatments to lower ATRA doses and decrease toxicity.

Natural compounds with anticancer effects are emerging as promising options to supplement traditional leukemia treatments. Bee venom (BV), produced by *Apis mellifera* L., contains numerous bioactive substances, with melittin accounting for approximately 50% of its dry weight [17]. Melittin is a 26-amino acid amphipathic peptide that exhibits strong anticancer activity through mechanisms different from retinoid-based differentiation therapy [18,19].

Critically, melittin induces apoptosis through calcium-dependent activation of the mitochondrial pathway, completely independent of retinoid receptors or differentiation signaling [20]. This mechanism offers several theoretical advantages for APL treatment: (1) it could be effective in ATRA-resistant cases, (2) it may bypass the molecular pathways involved in RAS development, and (3) it could potentially be combined with lower ATRA doses to minimize toxicity while maintaining effectiveness.

Previous research has demonstrated melittin’s effectiveness against various types of blood cancer. Our earlier findings revealed that melittin induces apoptosis specifically in acute lymphoblastic leukemia and chronic myelogenous leukemia cells by disrupting mitochondrial function and activating caspases, with minimal damage to healthy peripheral blood mononuclear cells [21]. Similar mitochondria-dependent mechanisms have been observed in other cancer types, suggesting a shared anticancer pathway that may be especially beneficial in leukemia therapy [22,23].

Building on melittin’s known anticancer effects in other leukemia types, this study investigates its impact on APL to see if it activates similar mitochondrial-dependent mechanisms in promyelocytic HL-60 model (PML/RARα-negative). Given the unique treatment challenges in APL—particularly ATRA resistance and RAS—understanding how melittin works in this setting could offer valuable insights for developing therapies that do not rely on retinoids.

The main objectives of this study were to: (1) evaluate the cytotoxic and pro-apoptotic effects of melittin on promyelocytic HL-60 model (PML/RARα-negative), (2) investigate the molecular mechanisms behind melittin-induced cell death, focusing on mitochondrial pathway involvement, and (3) analyze gene expression changes in key apoptotic regulators to better understand pathway specificity. These results will contribute to the growing evidence supporting melittin as a potential therapeutic agent and may inform future combination therapy strategies for APL treatment.

## 2. Results

### 2.1. Apoptosis by Annexin V

Dose selection for mechanistic assays was guided by concentration–response viability data; sub-cytotoxic and near-IC50 levels were used to delineate apoptosis and mitochondrial involvement.

**Figure 1:**
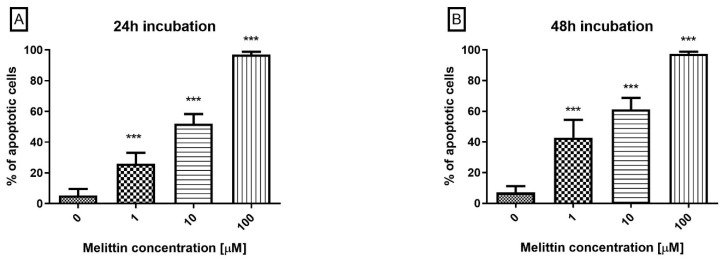
The effect of melittin on apoptosis induction in HL-60 cells. Apoptosis was measured by flow cytometry using Annexin V-propidium iodide staining after 24 h (**A**) and 48 h (**B**) of incubation with the toxin. The percentage of apoptotic cells was shown as a percentage of Annexin V^+^ objects in the tested sample. Values are means ± SD (*n* = 4). *** *p* < 0.001.

To determine the mode of cell death, Annexin V/propidium iodide (PI) co-staining by flow cytometry was used to analyse melittin-treated HL-60 cells. Co-staining enabled discrimination of apoptotic (Annexin V^+^), necrotic (PI^+^), and viable (Annexin V^−^/PI^−^) fractions. Across 24–48 h, Annexin V positivity increased in a dose- and time-dependent manner (Figure 1), approaching ~100% at 100 μM. Primary necrosis remained low (≤5% across conditions), indicating that apoptosis is the predominant mode of cell death under the tested conditions. This apoptosis-dominant profile aligns with prior reports in leukemic models.

### 2.2. Mitochondrial Depolarization (ΔΨM)

To link the apoptosis-dominant profile with the intrinsic pathway, mitochondrial transmembrane potential (ΔΨM) was quantified using JC-1. Loss of ΔΨM is a hallmark of mitochondrial permeabilization and precedes executioner caspase activation.

**Figure 2:**
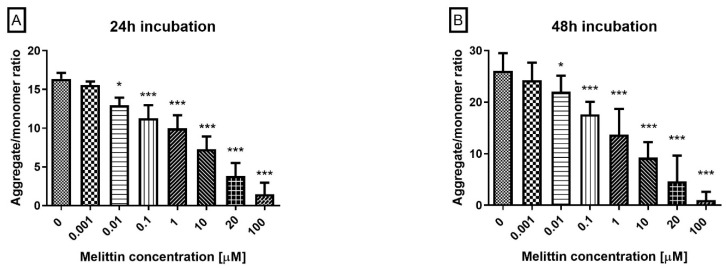
Effect of melittin on mitochondrial membrane potential (MMP) in HL-60 cells. Fluorescence was measured after 24 h (**A**) and 48 h (**B**) of incubation. In healthy, polarized mitochondria, JC-1 accumulates and forms aggregates that emit red fluorescence. In apoptotic cells, where mitochondrial depolarization occurs, JC-1 remains in its monomeric form in the cytoplasm, emitting green fluorescence. Data are shown as mean ± SD (*n* = 4). * *p* < 0.05; *** *p* < 0.001 compared to the control.

Melittin induced a concentration-dependent decrease in ΔΨM in HL-60 cells at 24 h and 48 h across the tested range (0.1–100 μM; Figure 2). Compared with vehicle, ΔΨM was significantly reduced at each concentration, with greater loss at higher doses and at 48 h. The shift in the JC-1 signal (lower red/green ratio) indicates progressive mitochondrial depolarization, consistent with the Annexin V apoptosis findings and supportive of intrinsic pathway engagement.

### 2.3. Caspase-3/7 Activity

**Figure 3:**
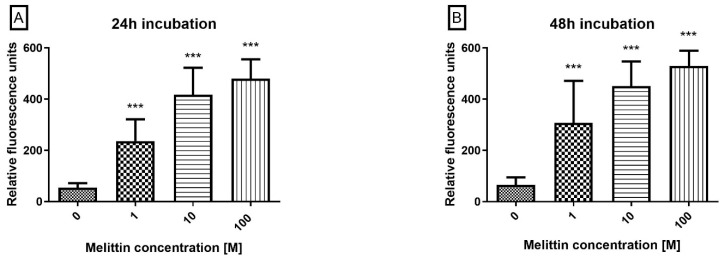
The effect of melittin on caspases 3/7 pathway activation in the HL-60 cell line. Caspase proteolytic activity was measured by flow cytometry; excitation 488 nm after 24 h (**A**) and 48 h (**B**) of incubation with toxin. Values are means ± SD (*n* = 4). *** *p* < 0.001.

To position the execution phase downstream of mitochondrial depolarization, caspase-3/7 enzymatic activity was quantified by flow cytometry after incubating cells with a fluorogenic DEVD-FMK (FAM) probe. Melittin produced a concentration-dependent increase in caspase-3/7 activity at 24 h and 48 h across 0.1–100 μM (Figure 3). Activity was significantly elevated at all tested concentrations (*p* < 0.001), with stronger activation at higher doses and after 48 h. These data, together with Annexin V positivity and ΔΨM loss, substantiate a caspase-dependent, apoptosis-dominant mechanism in HL-60 cells.

### 2.4. CASP8/CASP9 mRNA Levels

**Figure 4:**
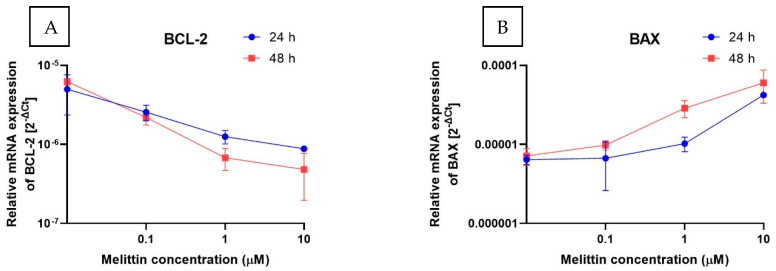

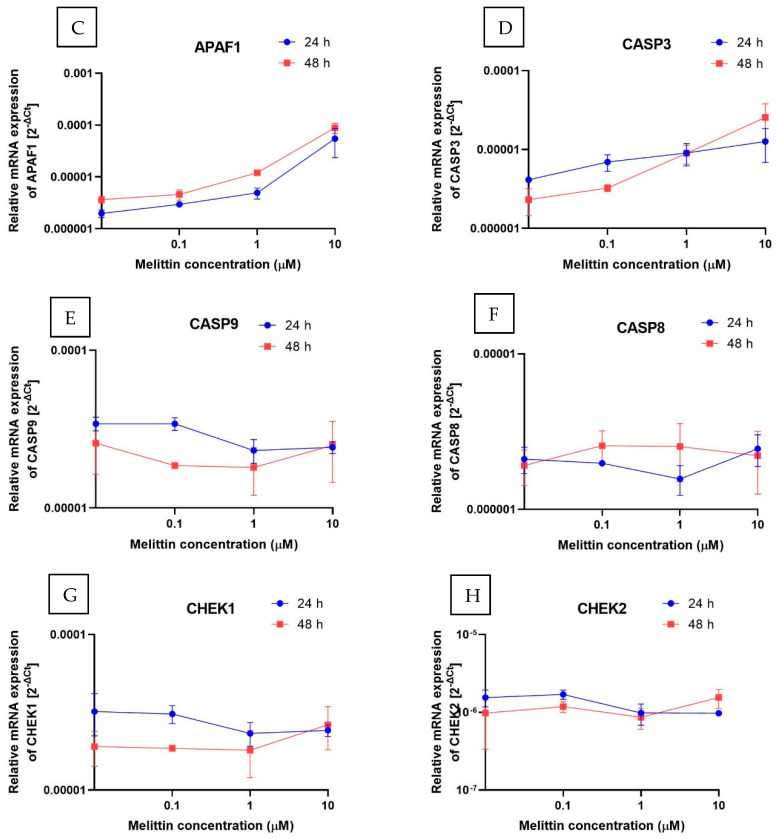
The effect of melittin on the expression of selected genes: (**A**) BCL-2, (**B**) BAX, (**C**) APAF1, (**D**) CASP3, (**E**) CASP9, (**F**) CASP8, (**G**) CHEK1, (**H**) CHEK2 (measured at the mRNA level) in HL-60 cell lines. The results are shown as the mean of 2^−ΔCt^ (based on the reference gene, 18S rRNA) ± SD, *n* = 4.

To complement the functional readouts, transcript levels of apoptosis-related genes were quantified by RT-qPCR (2^−ΔΔCt^). Three gene sets were analyzed: intrinsic-pathway regulators (BCL2, BAX, APAF1), caspases (CASP3, CASP8, CASP9), and cell-cycle checkpoints (CHEK1, CHEK2) (Figure 4). Across the tested conditions, BCL2 decreased in a concentration-dependent manner, whereas BAX and APAF1 increased, consistent with engagement of the intrinsic (mitochondrial) pathway. CASP3 mRNA was significantly upregulated, in line with the rise in caspase-3/7 enzymatic activity. By contrast, the transcripts for CASP8 and CASP9 remained essentially unchanged. Because caspase activation is predominantly post-translational, unchanged CASP8/9 mRNA does not preclude proteolytic activation. Taken together with Annexin V positivity and ΔΨM loss, this transcriptional profile supports a caspase-dependent, apoptosis-dominant mechanism of melittin action in HL-60 cells.

### 2.5. Cell Viability

**Figure 5:**
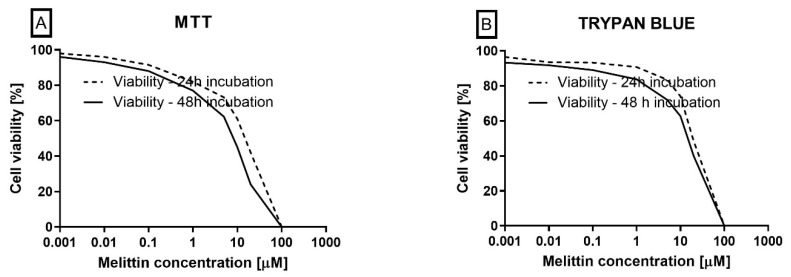
The effect of melittin (at concentrations ranging from 0.001 to 100 µM) on the viability of HL-60 cells. Cell viability was evaluated using the MTT (**A**) and trypan blue (**B**) methods. The data represent cell viability curves derived from four independent measurements (*n* = 4).

To contextualize dose selection for the mechanistic assays, cell viability was quantified using trypan blue exclusion and the MTT reduction assay. Melittin decreased viability in a concentration-dependent manner in both readouts (Figure 5). From the cytotoxicity curves, LC50 values were estimated at 20.21 μM (24 h) and 9.60 μM (48 h) for MTT, and 23.93 μM (24 h) and 13.99 μM (48 h) for trypan blue. These data provided the basis for selecting sub-cytotoxic and near-LC50 concentrations used in the apoptosis (Annexin V), ΔΨM, and caspase-3/7 activity experiments, supporting the apoptosis-dominant mechanism observed in HL-60 cells.

## 3. Discussion

Melittin, the primary bioactive peptide in bee venom, has been widely researched in recent years due to its cytotoxic and proapoptotic properties. Many studies have verified its anticancer potential across various malignancies, including breast and prostate cancer, melanoma, bladder, liver cancer, and leukemia [20,21,22,24]. Our study’s findings align with these observations, showing that melittin also produces strong cytotoxic and proapoptotic effects against promyelocytic HL-60 model (PML/RARα-negative).

While this study focused exclusively on a promyelocytic HL-60 model without evaluating non-cancerous hematopoietic controls, this approach reflects the standard methodology in natural product mechanistic research, where target cell characterization comes before selectivity testing. The therapeutic selectivity of melittin has been extensively documented across various cancer types, including our prior demonstration of minimal toxicity to healthy PBMCs at concentrations highly toxic to leukemia cells [25]. This pattern of selectivity has been independently confirmed by Gasanoff et al., who reported three times greater toxicity toward Jurkat leukemia cells compared to normal lymphocytes [26]. It is mechanistically linked to fundamental differences in membrane composition and mitochondrial vulnerability between cancer and normal cells [27,28]. In the context of APL, the key therapeutic significance is not just general selectivity—which is well understood—but rather in providing retinoid-independent mechanisms to overcome ATRA resistance. Our demonstration that melittin acts through entirely different mitochondrial pathways directly addresses this clinical need. It provides a solid mechanistic basis for moving forward to the next crucial research steps: assessing in vivo efficacy and optimizing combination therapies with existing APL treatments.

In our experiments, melittin decreased cell viability in a dose- and time-dependent manner, with LC_50_ values in the micromolar range. Similar effects were reported by Hait et al., who showed that melittin inhibits leukemia cell proliferation through calmodulin-related mechanisms [29]. Their pioneering studies, conducted nearly four decades ago, still form the basis for current research on melittin as a potential anticancer agent. Our findings agree with these observations and further clarify the mechanism of action, emphasizing melittin’s impact on mitochondrial integrity and the activation of the caspase cascade. Comparable effects in hematological malignancies were reported by Ceremuga et al., who examined melittin’s activity against ALL and CML cell lines. They found that melittin induces apoptosis via mitochondrial and caspase-dependent pathways while exerting minimal toxicity toward healthy PBMCs [25]. Our results confirm this reproducibility in the APL model, suggesting that melittin’s proapoptotic mechanism is consistent across various leukemia types, including both lymphoid and myeloid.

Similar anticancer properties of melittin have also been documented in chronic myeloid leukemia (CML). Halici et al. showed that low doses of bee venom—rich in melittin, apamin, secapin, phospholipase A2, and hyaluronidase—induce a strong, dose- and time-dependent antiproliferative effect in K-562 CML cells. The highest concentration tested (0.4 µM) reduced cell viability by 55%, 80%, and 92% after 24, 48, and 72 h, respectively. They also observed morphological changes, such as cell shrinkage and membrane degeneration, which indicate stress and induction of apoptosis [30]. Additionally, Obeidat et al. demonstrated that melittin levels in raw bee venom vary seasonally, with the highest concentration occurring in spring. In K-562 cells, both crude bee venom extract and purified melittin exerted cytotoxic effects, causing apoptosis, necrosis, and cell cycle arrest. Melittin primarily induced late apoptosis and moderate G0/G1 arrest, accompanied by an increase in cells at the G2/M phase. Furthermore, inhibition of the NF-κB/MAPK14 axis and downregulation of c-MYC and CDK4 were observed, along with a significant induction of ABL1, JUN, and TNF [31]. These findings confirm that melittin’s activity in CML involves both cytotoxic effects and gene regulatory mechanisms related to apoptosis and cell cycle control.

The apoptotic mechanisms observed in our study are consistent with those previously reported. We showed that melittin causes a significant depolarization of the mitochondrial membrane potential (ΔΨM), a key feature of intrinsic apoptosis. Similar mitochondrial dysfunction was noted by Othon et al., who found that melittin destabilizes biological membranes, disturbs calcium levels, and leads to mitochondrial breakdown [32]. Gasanoff et al. reported that melittin markedly disrupts mitochondrial respiration in both healthy lymphocytes and cancer cells (Jurkat), with cytotoxicity three times higher in tumor cells. This effect was linked to direct interactions with mitochondrial inner membranes and the formation of non-bilayer lipid structures, ultimately impairing cellular bioenergetics and causing cell death [26]. Our results support the idea that mitochondria are a primary target of melittin in cancer cells. The activation of caspases 3/7 we observed further confirms that melittin initiates the execution phase of apoptosis. At the gene expression level, we saw decreased BCL-2 and increased BAX and APAF1, consistent with the classical mitochondrial pathway.

Our gene expression analysis focused on key apoptotic regulators representing major pathway components: the intrinsic pathway (BCL-2, BAX, APAF1), executioner caspases (CASP3), and initiator caspases (CASP8, CASP9). Although this panel is targeted rather than comprehensive, these genes provide critical insights into the dominant apoptotic mechanisms. The lack of transcriptional changes in CASP8 and CASP9, along with the significant upregulation of intrinsic pathway components, suggests that the mitochondrial pathway prevails. However, we acknowledge that protein-level validation is necessary to definitively exclude involvement of the death receptor pathway, as post-translational modifications, protein cleavage, and enzymatic activation can occur independently of mRNA changes. Our conclusion regarding pathway preference is therefore based on multiple complementary approaches, including mitochondrial membrane potential loss, specific gene expression patterns, and caspase-3/7 enzymatic activity, rather than gene expression alone. This multi-parameter approach aligns with established methodologies in apoptosis research, where converging evidence from functional and molecular assays provides mechanistic insights. Importantly, our findings align with extensive literature demonstrating melittin’s preferential activation of intrinsic apoptosis across various cancer types [26,33], indicating that our observed pathway pattern reflects the well-established mechanism rather than an APL-specific deviation.

Importantly, we did not observe significant changes in CASP8 or CASP9 expression, suggesting that the death receptor pathway is not critically involved in HL-60 cells treated with melittin. Similar results were reported in solid tumors, where melittin mainly activated the mitochondrial pathway [28]. This supports the idea that mitochondria are the primary target of melittin in cancer cells. Comparable findings in myeloid leukemias were reported by Halici et al. and Obeidat et al., showing that both purified melittin and bee venom extracts significantly reduced K-562 cell survival by inducing apoptosis and cell cycle arrest, along with regulation of apoptosis- and proliferation-related genes such as ABL1, JUN, TNF, c-MYC, and CDK4 [30,31]. Kim et al. further showed that in apoptosis-resistant fibroblasts, melittin worked through the mitochondrial pathway, increasing the levels of caspase-3, caspase-9, Apaf-1, and cytochrome c, without activating the death receptor pathway (CASP8) [33]. Overall, these findings suggest that melittin’s mitochondrial mechanism is common and important across different cell types, both cancerous and non-cancerous, further supporting our observations.

Our findings confirm the known pattern of melittin’s anticancer activity by demonstrating consistent mitochondrial-dependent apoptosis in the promyelocytic HL-60 model. The observed cytotoxic effects, mitochondrial membrane depolarization, caspase-3/7 activation, and changes in pro-apoptotic gene expression closely align with previous reports in other leukemia types, supporting the idea that melittin’s mechanism operates across hematological cancers. These results are consistent with retinoid-independent apoptosis; validation in PML/RARα-positive APL (e.g., NB4) is a logical next step. The consistent mechanism observed in ALL, CML, and now APL provides strong evidence for melittin’s potential as a universal leukemia therapy, especially given the different molecular origins of these leukemia types.

It is worth noting that melittin’s anticancer effects are not limited to hematological malignancies. Son et al. described the pleiotropic activity of bee venom and its components, showing that melittin can induce apoptosis across a wide range of tumors, including breast, liver, lung cancer, and melanoma [27]. In breast cancer, Zhou et al. demonstrated that melittin enhances the sensitivity of tumor cells to standard cytostatics, suggesting a potential synergistic effect [14]. Similarly, studies on hepatocellular carcinoma have shown that melittin promotes apoptosis while decreasing the invasive and metastatic capabilities of the disease [34]. A recent meta-analysis by Prince et al. confirmed melittin’s potential in breast cancer treatment: the analysis of 40 studies revealed that melittin induces cytotoxicity, apoptosis, and gene regulation, destabilizes cancer cell membranes, and modulates key apoptotic pathways. Advanced delivery systems have been shown to improve their effectiveness and lower toxicity, while combination therapy with chemotherapy has demonstrated synergistic effects and better therapeutic results [35]. Moreover, Ye and Lei reported that melittin significantly reduces the viability of hepatoma cells (Huh7 and HepG2) in a dose- and time-dependent manner, with proteomic analysis revealing changes in the expression levels of many proteins. HCS assays indicated that inhibition of LARS2 and ZNF19 contributed to decreased tumor cell proliferation, suggesting a molecular mechanism behind melittin’s anti-hepatoma activity [36]. Our findings of strong caspase activation in HL-60 cells align with this model, implying that melittin may also lower the apoptotic induction threshold in leukemias, thereby boosting the effectiveness of conventional drugs. Overall, these data highlight the universal nature of melittin’s proapoptotic mechanism across different cancer types and its potential for use in combination therapy.

Our findings identify melittin as an inducer of mitochondrial, caspase-dependent apoptosis in HL-60 cells, consistent with recent evidence that melittin causes ΔΨM loss and activates executioner caspases across hematologic and solid-tumor models. The apoptosis-dominated responses observed here align with previous reports in U937 leukemia and other cell lines, where Annexin V positivity and caspase-3 activation are predominant, and primary necrosis mainly occurs at supra-cytotoxic exposures [37,38]. Additionally, recent studies suggest that melittin can reduce classic multidrug resistance (MDR) phenotypes and serve as a chemosensitizer, decreasing resistance in certain models [39,40]. Collectively, our results with HL-60 cells support this literature: melittin activates the intrinsic apoptotic pathway with minimal primary necrosis within the tested range, and its potential to modulate MDR provides a plausible basis for further translational research. Consistent with the scope of this work, we limit our conclusions to within-compound effects in HL-60 and have updated the reference list to include these recent contributions.

However, current evidence suggests that melittin can mitigate classical multidrug resistance (MDR) phenotypes rather than readily inducing them. Because its primary cytotoxicity stems from rapid membrane perturbation and downstream mitochondrial apoptosis, melittin operates largely outside canonical efflux-based resistance; in several models, it also acts as a chemosensitizer, restoring responsiveness to cytotoxics (e.g., cisplatin) via suppression of stress-adaptive programs such as HSF1-driven glycolytic signaling and through formulation-enabled co-delivery strategies that overcome MDR [41]. At the same time, adaptive tolerance cannot be excluded: susceptibility to melittin depends on membrane lipid composition, with cholesterol and raft organization dampening pore formation and thereby modulating sensitivity [42]. Overall, available data suggest low propensity for classic P-gp-mediated resistance and a meaningful potential for melittin to reduce resistance burden as part of rational combinations [43].

From the perspective of APL therapy, it is particularly relevant to compare our results with standard treatments such as ATRA and anthracyclines. Lo-Coco and Hasan, as well as Sanz et al., emphasized that combining ATRA with anthracyclines leads to complete remission in most patients; however, challenges remain due to retinoid syndrome and the development of resistance [21,44]. Our study suggests that melittin acts via a distinct mechanism, independent of the retinoic acid receptor, making it a potential adjuvant candidate in ATRA-resistant patients. Similarly, Zhou et al. reported improved APL treatment outcomes with combination therapy (ATRA + ATO), highlighting the possibility of exploring melittin in differentiation-based regimens [14].

A key aspect of melittin’s mechanism is its ability to interact with cell membranes. Othon et al. showed that melittin undergoes dynamic conformational transitions between monomeric and tetrameric forms, enhancing its affinity for biological membranes [32]. This effect is particularly pronounced in cancer cells, which exhibit an altered lipid composition and increased exposure of phosphatidylserine on their surface. Our findings—loss of mitochondrial membrane potential (ΔΨM) and increased BAX and APAF1 expression—indicate that mitochondria are the primary target of melittin, consistent with earlier reports by Gasanoff et al. and with caspase 3/7 activation in HL-60 cells.

Another critical aspect is melittin’s potential synergy with standard therapies. Stahl and Tallman emphasized that despite progress in APL treatment, some patients still experience incomplete responses or relapses [13]. Our data suggest that melittin, by enhancing mitochondria-mediated apoptosis, could complement ATRA or anthracyclines at lower doses, reducing toxicity risks. The synergistic effects of melittin have also been confirmed in other cancers, where its combination with chemotherapeutics (e.g., doxorubicin, gemcitabine, 5-FU) significantly enhances efficacy and reduces multidrug resistance [45,46,47,48].

An important consideration for the clinical translation of melittin in APL treatment is addressing its bioavailability and bioaccessibility challenges. Melittin’s systemic toxicity, hemolytic activity, and potential for allergic reactions present significant pharmacokinetic limitations that must be overcome for therapeutic application. However, extensive research has already established multiple promising strategies to address these challenges. Advanced delivery systems, including pH-sensitive modifications such as MLT-DMMA that activate specifically in acidic tumor microenvironments, peptide-drug conjugates like M-DM1 that selectively target tumor-associated macrophages, and sophisticated carrier-based approaches using polymersomes, liposomes, and functionalized nanoparticles, have demonstrated the ability to enhance melittin’s therapeutic index while minimizing systemic toxicity [35,49]. Additionally, combination strategies using natural compounds have shown synergistic effects, protecting healthy cells from peptide-induced toxicity. While bioavailability optimization remains a critical translational challenge, our demonstration of melittin’s retinoid-independent mitochondrial mechanism in promyelocytic HL-60 model (PML/RARα-negative) provides the essential mechanistic foundation for rational design of these delivery approaches [40,50,51]. The consistent efficacy of this mechanism across different leukemia subtypes, combined with the established selectivity for cancer cells over normal cells, supports continued investigation of optimized melittin formulations as a complementary therapeutic strategy for APL, particularly in ATRA-resistant cases where conventional treatment options are limited.

Given its systemic toxicity, hemolytic activity, and risk of allergic reactions, several strategies are being developed to improve melittin’s safety. Promising approaches include chemical and physicochemical modifications of the peptide, such as pH-sensitive amide linkers (MLT-DMMA), which activate only in the acidic tumor microenvironment [52], peptide–drug conjugates like M-DM1, which selectively target M2-like tumor-associated macrophages while enhancing effector cell infiltration [49], as well as hybrid peptides combining melittin fragments with other bioactive sequences to improve anticancer efficacy while limiting toxicity to normal cells [53,54]. Alternative strategies include carrier-based delivery systems, such as polymersomes, liposomes, and functionalized nanoparticles, which enable the controlled co-delivery of melittin and chemotherapeutics (e.g., doxorubicin), thereby enhancing anticancer efficacy and minimizing side effects [39,45,52,55]. Furthermore, combining melittin with natural compounds (e.g., sericin, EGCG, curcumin) has shown synergistic antiproliferative and proapoptotic effects, while protecting healthy cells from peptide-induced toxicity [53,56].

Looking ahead, Al-Hamaly et al. demonstrated that mitochondrial modulation in T-ALL cells may increase the sensitivity of resistant populations, suggesting that melittin could help eliminate resistant leukemia cells [55]. Zhang et al. further showed that melittin can stimulate Schwann cell proliferation under hyperglycemic conditions via the Crabp2/Wnt/β-catenin pathway, highlighting the context-dependent selectivity of melittin’s action, which may have implications for both targeted cancer and regenerative therapies [57].

In summary, our findings confirm the strong cytotoxic and proapoptotic effects of melittin against acute promyelocytic leukemia cells. The consistency of these results with previous studies in other leukemias and solid tumors underscores the universal nature of melittin’s mechanism of action. At the same time, its distinction from classical APL therapies suggests that melittin could serve as a potential adjuvant in combination regimens. Further in vivo studies and the development of delivery systems are essential to evaluate the clinical applicability of melittin.

## 4. Materials and Methods

### 4.1. Reagents

Dimethyl sulfoxide (DMSO), Tris buffer, Histopaque^®^-1077, RPMI 1640 medium, (S)-(+)-camptothecin (C9911), and melittin derived from honeybee venom (catalog no. M2272) were obtained from Sigma-Aldrich Chemical Co. (St. Louis, MO, USA). The cell viability kit, which included counting slides and trypan blue dye, was supplied by BIO-RAD (Hercules, CA, USA). Penicillin–streptomycin solution, heat-inactivated fetal bovine serum (FBS), and phosphate-buffered saline (PBS) without calcium and magnesium were purchased from Lonza (Basel, Switzerland). JC-1 dye, MTT reagent [3-(4,5-dimethylthiazol-2-yl)-2,5-diphenyltetrazolium bromide], Hank’s Balanced Salt Solution (HBSS), and the CellEvent™ Caspase-3/7 Green Flow Cytometry Assay Kit were provided by Thermo Fisher Scientific (Waltham, MA, USA). The Annexin V FITC Apoptosis Detection Kit I was sourced from Becton Dickinson (Franklin Lakes, NJ, USA). All other reagents used were of molecular biology grade.

### 4.2. Cell Culture

The human leukemia cell line HL-60 was purchased from the American Type Culture Collection (ATCC™, Manassas, VA, USA) and cultured in RPMI 1640 medium supplemented with 10% (*v*/*v*) fetal bovine serum (FBS), 100 U/mL penicillin, and 100 μg/mL streptomycin sulfate. The cells were maintained in an exponential growth phase under a humidified atmosphere of 5% CO_2_ at 37 °C. For the experiments, HL-60 cells (3 × 10^6^ cells/well) were pre-incubated for 12 h and subsequently treated with melittin at concentrations ranging from 0.001 to 100 µM for 24 and 48 h. A pharmacological positive control was not included in this dataset; assay qualification relied on vehicle- and time-matched controls, as well as melittin concentration–response.

### 4.3. Cell Viability Determination

The viability analysis of melittin-treated HL-60 cells was performed using two independent methods: the MTT assay and a cell counter with trypan blue dye.

The MTT assay was performed by adding MTT solution (0.5 mg/mL) to all samples, followed by incubation at 37 °C for 4 h. After incubation, the MTT solution was carefully removed, and the resulting formazan crystals were dissolved in dimethyl sulfoxide (DMSO). The quantity of formazan crystals was determined colorimetrically using a microplate reader (BioTek Synergy HT, BioTek Instruments, Winooski, VT, USA) at a primary wavelength of 570 nm with background subtraction at 630 nm. Cell viability was calculated as a percentage relative to the untreated control cells, which were assigned a value of 100%.

As a second assessment, cell viability was evaluated using the trypan blue exclusion method with the BIO-RAD TC20™ Automated Cell Counter (Hercules, CA, USA), following the manufacturer’s protocol. Cell viability was expressed as a percentage relative to the untreated control cells, which were defined as 100%.

### 4.4. Mitochondrial Membrane Potential (MMP)

Mitochondrial membrane potential (MMP) serves as a crucial marker for assessing mitochondrial function, which is tightly linked to overall cellular health. To analyze MMP, we employed the fluorescent, membrane-permeable dye JC-1 (5′,6,6′-tetrachloro-1,1′,3,3′-tetraethylbenzimidazolylcarbocyanine iodide).

Initially, cells were seeded into 96-well black microplates with clear bottoms suitable for fluorescence detection (Greiner Bio-One, Kremsmünster, Austria) at a density of 1 × 10^5^ cells per well in 50 µL of culture medium. Following a 12 h period, the cells were processed in suspension with melittin according to the protocol described previously.

Subsequently, JC-1 dye was applied at a final concentration of 5 µM (prepared in HBSS), and the cells were incubated for 30 min at 37 °C in a humidified incubator with 5% CO_2_. After staining, the cells were washed twice with HBSS, followed by centrifugation at 300× *g* for 10 min at 22 °C.

Fluorescence intensity was then measured using a Bio-Tek Synergy HT microplate reader (Bio-Tek Instruments, Winooski, VT, USA) with dual excitation/emission filter sets: 485 nm/538 nm for monomers and 530 nm/590 nm for aggregates. MMP was expressed as the ratio of red (aggregate) to green (monomer) fluorescence.

### 4.5. Apoptosis Assay–Annexin V Binding

Apoptotic cells were identified through detection of phosphatidylserine exposure using flow cytometry with the FITC Annexin V Apoptosis Detection Kit I (BD Pharmingen™, Franklin Lakes, NJ, USA). Following the culture procedure, cells were washed twice with cold PBS and resuspended in 100 μL of 1× annexin-binding buffer (cell density: 1 × 10^5^) in cytometry tubes. Subsequently, 5 µL of FITC-conjugated Annexin V and 5 µL of propidium iodide (PI) were added to each sample. The mixtures were then incubated at 37 °C in a humidified environment containing 5% CO_2_ for 15 min.

After incubation, 400 μL of 1× annexin-binding buffer was added to each tube, and the samples were subjected to flow cytometric analysis using a PARTEC CUBE 6 cytometer (Görlitz, Germany) with 488 nm excitation. Fluorescence detection was performed using a 536/40 nm bandpass filter for FITC and a 630 nm longpass filter for PI. Signal gates were established based on the fluorescence profiles of unstained control samples. The apoptotic index was determined by calculating the mean percentage of FITC-positive cells among 5 × 10^4^ analyzed cells per experiment. Flow cytometric data were processed using CyFlow software, version 1.5.1.2.

### 4.6. Determining the Activity of Caspase-3/Caspase-7

Caspase-3/7 activity was assessed using the CellEvent™ Caspase-3/7 Green Flow Cytometry Assay Kit. Following cell culture, the cells were rinsed twice with cold PBS and resuspended in 1 mL of PBS at a concentration of 1 × 10^5^ cells per cytometric tube. Subsequently, 1 µL of the CellEvent™ Caspase-3/7 Green Detection Reagent was added, and the suspension was gently mixed. The samples were then incubated for 30 min at 37 °C in a humidified 5% CO_2_ atmosphere. Following this, 1 µL of SYTOX™ AADvanced™ dead cell stain (prepared at 1 mM in DMSO) was added to each tube and incubated for an additional 5 min at room temperature in the dark.

Without any further washing or fixation steps, fluorescence was measured using a Bio-Tek Synergy HT Microplate Reader (Bio-Tek Instruments, Winooski, VT, USA), with dual filter settings of 530 nm/590 nm and 485 nm/538 nm.

### 4.7. Gene Expression Analysis Using Real-Time PCR

Total RNA was isolated from cell pellets using the EXTRACT ME RNA and DNA Kit (BLIRT S.A., Gdańsk, Poland), following the manufacturer’s instructions. The purity and concentration of the extracted RNA were assessed by measuring absorbance at 260 and 280 nm with a Bio-Tek Synergy HT Microplate Reader (Bio-Tek Instruments, Winooski, VT, USA). RNA samples were stored at −30 °C until subsequent analysis. Complementary DNA (cDNA) was synthesized from total RNA using the High-Capacity cDNA Reverse Transcription Kit, with 2 μg of RNA employed as template in a final reaction volume of 10 μL. Gene expression levels were quantified by real-time PCR employing the TaqMan probe-based detection system using the following commercially available TaqMan^®^ gene expression assays: BAX (Hs00180269_m1), BCL-2 (Hs00608023_m1), CAS-3 (Hs00234387_m1), CAS-8 (Hs01018151_m1), CAS-9 (Hs00962278_m1), APAF-1 (Hs00559441_m1), CHEK1 (Hs00967510_g1), CHEK2 (Hs00200485_m1) and 18S rRNA (Hs99999901_s1) as a reference gene.

Reactions were performed using a CFX96™ Real-Time PCR Detection System (Bio-Rad Laboratories, Hercules, CA, USA) [58]. The thermal profile consisted of an initial polymerase activation step at 95 °C for 10 min, followed by 40 cycles of denaturation at 95 °C for 30 s and annealing/extension at 60 °C for 1 min. Each sample was analyzed in triplicate. Threshold cycle (Ct) values were determined automatically by the CFX96 system using CFX Manager software (version 3.1). Relative gene expression levels were calculated according to the 2^−ΔCt^ method, where ΔCt = Ct of the target gene − Ct of 18S rRNA.

### 4.8. Data Analysis

All experimental data were processed using Microsoft Excel version 2508 (build 19127.20302) (Redmond, WA, USA) and are presented as means ± standard deviation (SD). Statistical analyses were conducted with the aid of StatsDirect software, version 2.7.2 (Cheshire, UK). Initially, the distribution of each dataset was assessed for normality using the Shapiro–Wilk test. Homogeneity of variances was subsequently verified via Levene’s test. Depending on the outcome of these preliminary tests, statistical significance between groups was evaluated using either one-way ANOVA followed by Tukey’s post hoc test (for normally distributed data with equal variances) or the non-parametric Kruskal–Wallis test. A *p*-value below 0.05 was considered indicative of statistical significance [59,60,61].

## 5. Conclusions

This study confirms and expands on existing evidence of melittin’s anticancer activity by showing consistent mitochondrial-dependent apoptosis in acute promyelocytic leukemia cells. Our findings support the notion that melittin exhibits similar effects across various leukemia types, underscoring its potential as a complementary therapy in the treatment of APL. The retinoid-independent mechanism presented here suggests particular usefulness for ATRA-resistant cases or combination treatments designed to reduce toxicity. Rather than presenting a discovery, these results strengthen the basis for developing melittin-based therapies by showing consistent mechanisms across various blood cancers. Future research should include in vivo testing, optimizing delivery methods, and systematically studying combination strategies with current APL treatments to bring this proven anticancer agent closer to clinical use.

Additionally, future studies should thoroughly validate the apoptotic pathways identified in our gene expression analysis at the protein level. Specifically, Western blot analysis of cleaved caspase-8, caspase-9, and caspase-3, along with assessments of cytochrome c release and mitochondrial fractionation, would offer clear mechanistic insights. Furthermore, pathway-specific inhibition studies using Z-IETD-FMK (a caspase-8 inhibitor) and Z-LEHD-FMK (a caspase-9 inhibitor) could help determine the distinct roles of the extrinsic and intrinsic pathways. This validation is crucial for developing rational combination therapies. It would complement our current findings by confirming the pathway preferences indicated by our multi-parameter functional analysis at the protein level.

The clinical significance of these findings is not in discovering new anticancer mechanisms but in providing mechanistic validation for retinoid-independent therapies that could overcome current challenges in APL treatment, especially ATRA resistance and retinoic acid syndrome.

## Data Availability

The original contributions presented in this study are included in the article. Further inquiries can be directed to the corresponding author(s).

## Abbreviations

The following abbreviations are used in this manuscript:

9-cis RA	9-cis retinoic acid
APL	acute promyelocytic leukemia
allo-HSCT	allogeneic hematopoietic stem cell transplantation
AML	acute myeloid leukemia
ANOVA	analysis of variance
APAF1	apoptotic protease-activating factor 1
ATRA	all-trans retinoic acid
auto-HSCT	autologous hematopoietic stem cell transplantation
BAX	Bcl-2-associated X protein
BCL-2	B-cell lymphoma 2
BV	bee venom
CASP3	caspase-3
CASP8	caspase-8
CASP9	caspase-9
cDNA	complementary DNA
Ct	cycle threshold
DIC	disseminated intravascular coagulation
DMSO	dimethyl sulfoxide
ΔΨM	mitochondrial transmembrane potential
FBS	fetal bovine serum
FITC	fluorescein isothiocyanate
HBSS	Hank’s Balanced Salt Solution
HSCT	hematopoietic stem cell transplantation
JC-1	5′,6,6′-tetrachloro-1,1′,3,3′-tetraethylbenzimidazolylcarbocyanine iodide
MCDP	mast cell-degranulating peptide
MMP	mitochondrial membrane potential
MPTP	mitochondrial permeability transition pore
MRD	minimal residual disease
MTT	3-(4,5-dimethylthiazol-2-yl)-2,5-diphenyltetrazolium bromide assay
PBMCs	peripheral blood mononuclear cells
PBS	phosphate-buffered saline
PI	propidium iodide
PML	promyelocytic leukemia
PML/RARα	promyelocytic leukemia/retinoic acid receptor-alpha fusion protein
qPCR	quantitative polymerase chain reaction (real-time PCR)
RARα	retinoic acid receptor-alpha
RAS	retinoic acid syndrome
RT-PCR	reverse transcription polymerase chain reaction
SD	standard deviation
TxA	anthracycline-based chemotherapy
